# Case Report: Serum AKR1B10 as a complementary marker for the detection of AFP-negative hepatocellular carcinoma – a report of two cases

**DOI:** 10.3389/fmed.2026.1756950

**Published:** 2026-03-13

**Authors:** Ye Gao, Lihua Xie, Yuting Ou, Xi Zeng, Deliang Cao, Jianping Yin

**Affiliations:** 1 Hunan Engineering Research Center for Early Diagnosis and Treatment of Liver Cancer, Hunan Province Key Laboratory of Tumor Cellular & Molecular Pathology, Cancer Research Institute, Hengyang Medical School, University of South China, Hengyang, Hunan, China; 2 Center for Health Management, Department of Pathology, The First Affiliated Hospital, Hengyang Medical School, University of South China, Hengyang, Hunan, China

**Keywords:** AFP, AKR1B10, hepatocellular carcinoma, misdiagnosis, serum markers

## Abstract

Hepatocellular carcinoma (HCC) is a leading cause of cancer-related deaths worldwide, with chronic hepatitis B and C viral infections being major risk factors. To date, the lack of effective methods for monitoring the progression from viral infection to cancer remains a clinical issue. Alpha-fetoprotein (AFP) is a serum marker used for the detection of HCC; however, AFP levels remain negative in 30–40% of HCC cases and 60–70% of early-stage HCC cases. Aldo-keto reductase 1B10 (AKR1B10) has been reported as a serum marker for HCC. In this study, we report two cases of hepatitis B-related HCC that were persistently AFP-negative but showed a marked increase in serum AKR1B10 levels. These cases illustrate the complementary role of serum AKR1B10 in the diagnosis of AFP-negative HCC, serving as a hypothesis-generating approach. A prospective large-scale study is warranted to define the clinical value of AKR1B10 in AFP-negative HCC.

## 1 Introduction

Hepatocellular carcinoma (HCC) accounts for 85–90% of primary liver cancers and is associated with high incidence and mortality (1). The primary risk factors for HCC are hepatitis B virus (HBV) and hepatitis C virus (HCV) infections, followed by alcohol intake, obesity, and diabetes (2). Therefore, it is of utmost importance to develop effective methods for the early detection of HCC in patients with viral infections.

Alpha-fetoprotein (AFP) is a glycoprotein that plays an immunomodulatory role during fetal development. AFP is expressed in HCC, hepatoblastoma, and germ cell tumors, and it has been used as a serum marker for HCC for over 40 years (3). To date, serum AFP levels combined with abdominal ultrasonography has been the primary strategy for the screening and detection of HCC in high-risk populations (4); however, its diagnostic efficacy is limited, particularly for early-stage HCC. A meta-analysis indicated that AFP had a sensitivity of 44.4%, a specificity of 84.8%, and an area under the curve (AUC) value of 0.52 for the detection of early-stage HCC (5). In addition, AFP has been reported to exhibit false positivity under certain conditions, such as liver cirrhosis (6). Therefore, there is a need to develop novel serum markers for the screening and detection of early HCC and AFP-negative HCC.

Aldo-keto reductase 1B10 (AKR1B10) has been reported as a serum marker for HCC (7–9). In normal tissues, AKR1B10 is mainly expressed in the colon and small intestine; however, its levels are upregulated in HCC (10, 11). A large-scale, multicenter study demonstrated that serum AKR1B10 serves as a diagnostic marker for HCC and early-stage HCC (7). In combination with AFP, AKR1B10 markedly increased the detection rate of HCC.

This study reports two clinical cases of HCC related to hepatitis B virus infection, which were unfortunately missed or misdiagnosed. In these two cases, serum AFP levels remained persistently negative, while serum AKR1B10 levels were markedly elevated, suggesting a potential role for AKR1B10 in the diagnosis of AFP-negative HCC. By presenting these illustrative cases, we highlight a real-world challenge in the diagnosis of HCC and draw attention to AKR1B10 as an adjunct serum marker for AFP-negative HCC, thereby encouraging large-scale prospective studies.

## 2 Case reports

### 2.1 Case 1

**Figure 1:**
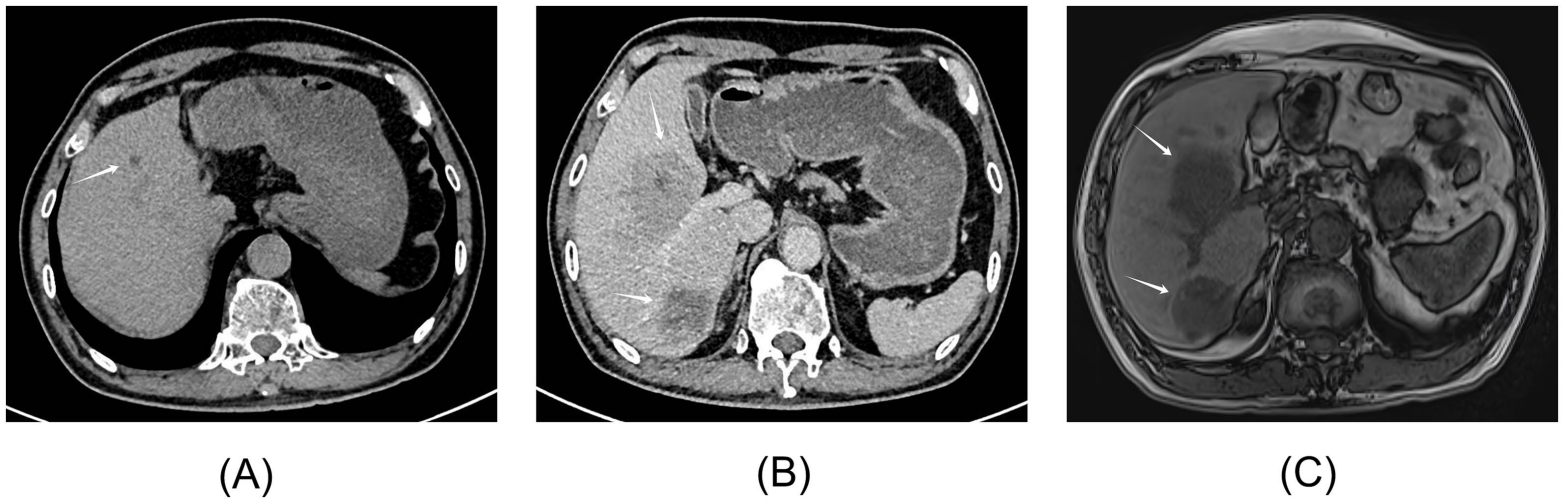
Imaging findings of Case 1. **(A)** Unenhanced and contrast-enhanced CT of the liver in September 2023. A low-density lesion of approximately 2.3 × 2.1 × 1.4 cm in the right lobe of the liver was considered an “atypical hepatic hemangioma.” **(B)** Contrast-enhanced CT of the upper abdomen in September 2024. Low-density, circular shadows with relatively clear boundaries were observed in liver segments S5 and S6. The larger lesion was approximately 8.8 × 8.2 cm. Enhanced scanning showed marginal enhancement of the lesion in the arterial phase and continuous enhancement of the lesion in the venous and delayed phases. **(C)** Upper abdominal MRI in September 2024, including unenhanced, contrast-enhanced, DWI sequences, and magnetic resonance cholangiopancreatography. Space-occupying lesions in liver segments S5 and S6 measured 10.0 × 8.0 cm and 4.4 × 3.8 cm, respectively. The lymph nodes in the hepatic portal area were enlarged.

In September 2024, a 68-year-old man with a 40-year history of chronic HBV infection visited the medical center for an annual physical examination. Unfortunately, he was diagnosed with advanced-stage HCC during this visit. The patient reported that he had undergone routine physical examinations since October 2021 and that this was his fourth annual visit (Table 1). He was asymptomatic during routine examinations. Serum AFP and carcinoembryonic antigen (CEA) levels were persistently negative during all visits, and liver function tests were normal in the previous visit. In October 2021, an abdominal ultrasound found several hepatic cysts, with the largest cyst measuring 1.4 × 1.1 cm. In August 2022, the patient underwent a second annual physical examination, which resulted in similar clinical and abdominal ultrasound findings. In September 2023, the patient underwent a third annual physical examination. In addition to the cysts, abdominal ultrasound of the liver revealed a mixed echoic nodule measuring 2.3 × 2.1 cm. The boundary of the nodule was unclear, and the internal echo was uneven. A few days later, computed tomography (CT) confirmed a lesion in the right lobe of the liver measuring approximately 2.3 × 2.1 × 1.4 cm, but this nodule was considered an “atypical hepatic hemangioma” (Figure 1A). The patient experienced no apparent discomfort and maintained a fair appetite.

**Table 1:** Summary of clinical visits, laboratory results, and imaging data for Case 1.

Time	AFP (ng/mL)	CEA (ng/mL)	AKR1B10 (pg/mL)	Liver function	Abdominal ultrasound	Enhanced CT	MRI
October 2021	Negative	Negative	N/A	Normal	Several cysts; the largest measuring 1.4 × 1.1 cm	N/A	N/A
August 2022	Negative	Negative	N/A	Normal	Several cysts; the largest measuring 1.6 × 1.2 cm	N/A	N/A
September 2023	Negative	Negative	N/A	Normal	Several cysts; the largest measuring 2.3 × 2.1 cm. An internal echo nodule with heterogeneous echoes and no detectable blood flow	A low-density lesion measuring 2.3 × 2.1 × 1.4 cm in the right hepatic lobe, indistinguishable from the liver parenchyma. An “atypical hepatic hemangioma.”	N/A
September 2024	Negative	Negative	6239.29	AST 75.90 U/L; AST/ALT 3.47	Two hyperechoic solid masses measuring 9.0 × 8.0 cm and 4.2 × 3.7 cm. Heterogeneous internal echoes with detectable blood flow	Low-density, circular shadows in liver segments S5 and S6 measuring 8.8 × 8.2 cm. Marginal enhancement in the arterial phase, and continuous enhancement in the venous and delayed phases.	Masses in liver segments S5 and S6, measuring 10.0 × 8.0 cm and 4.4 × 3.8 cm, respectively, with enlarged lymph nodes at the hepatic portal.

N/A, not available.

**Figure 2:**
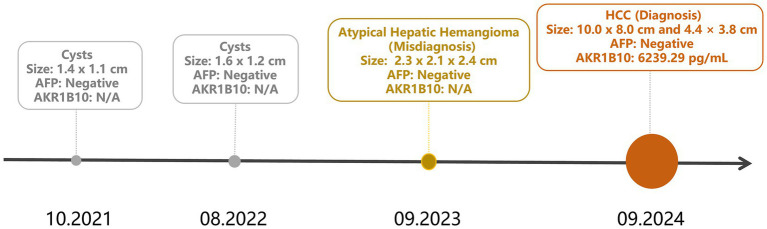
Timeline of clinical visits in Case 1. The infographic illustrates the chronological sequence of clinical visits and diagnoses. Circular markers are scaled proportionally to the lesion sizes. The details of the diagnostic findings are summarized in Table 1. N/A (not available) indicates that the AKR1B10 test was not performed at the indicated time point.

In September 2024, the patient presented for his fourth annual health visit with dull epigastric pain. He did not experience nausea, vomiting, chills, fever, chest tightness, shortness of breath, or other discomforts. The physical examination showed tenderness upon liver percussion. Laboratory tests revealed markedly elevated serum AKR1B10 levels at 6239.29 pg/mL (reference range: 0–369.30 pg/mL, as defined in the instruction), while AFP and CEA levels remained negative. Aspartate transaminase (AST) was 75.90 U/L, the AST/ALT ratio was 3.47, and urobilinogen (UBG) was 1.0 μmol/L. Abdominal ultrasound found two hyperechoic masses in the liver, measuring 9.0 × 8.0 cm and 4.2 × 3.7 cm. The boundaries of the masses were clear, and the internal echoes were uneven, with visible blood flow signals. The upper abdominal CT showed slightly low-density, circular shadows in liver segments S5 and S6, with relatively clear boundaries. The larger lesion measured approximately 8.8 × 8.2 cm (Figure 1B). Enhanced CT scans showed marginal enhancement of the lesion in the arterial phase and continuous enhancement of the lesion in the venous and delayed phases. Magnetic resonance imaging (MRI) found space-occupying lesions in liver segments S5 and S6, measuring 10.0 × 8.0 cm and 4.4 × 3.8 cm, respectively (Figure 1C). The lymph nodes in the hepatic portal area were also enlarged. The patient was clinically diagnosed with primary liver cancer involving the right branch of the portal vein. The diagnosis was confirmed as LR-5 (Definite HCC) based on the latest EASL Clinical Practice Guidelines and the LI-RADS v2018. The lesion exhibited non-rim arterial phase hyperenhancement, non-peripheral washout, and an enhancing capsule, which rendered invasive biopsy unnecessary. Figure 2 shows the timeline of clinical visits and the diagnostic history of Case 1.

### 2.2 Case 2

**Figure 3:**
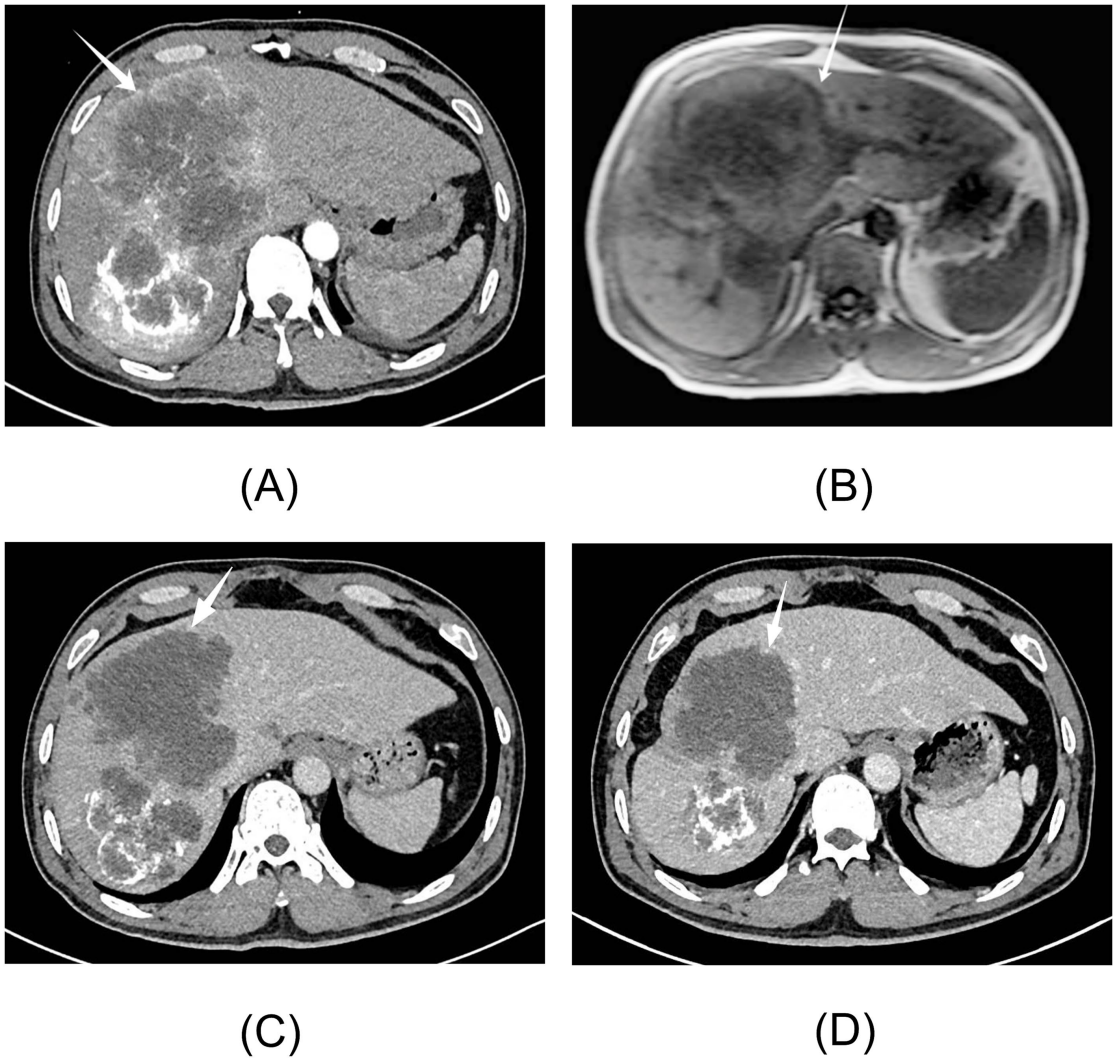
Imaging findings of Case 2. **(A)** Contrast-enhanced CT of the abdomen at initial diagnosis. An intrahepatic space-occupying lesion with a maximal cross-section of 18.1 × 13.1 cm was found. **(B)** Unenhanced and hepatocyte-specific contrast-enhanced liver MRI at initial diagnosis. An intrahepatic lesion with a maximal cross-section of 11.8 × 13.5 cm was found. **(C)** The tumor diameter was 10.8 × 9.6 cm after the first TACE. **(D)** The tumor diameter was 8.3 × 8.1 cm after the third TACE. TACE, transcatheter arterial chemoembolization.

In February 2025, a 49-year-old male patient with a history of chronic HBV infection for over 10 years visited the medical center. He reported nausea, acid regurgitation, and retching but denied any other gastrointestinal symptoms, such as abdominal pain, diarrhea, hematemesis, or melena. The physical examination upon admission was unremarkable. The abdomen was soft, with no stigmata of chronic liver diseases, such as palmar erythema or spider nevi. Laboratory tests revealed a serum AKR1B10 level of 1500.00 pg/mL (reference range: 0–369.30 pg/mL). AFP, CEA, and tumor-specific growth factor were within normal limits, measuring 5.67 ng/mL (reference range: 0–7.0 ng/mL), 1.19 ng/mL (reference range: 0–5 ng/mL), and 47.3 U/mL (reference range: 0–64 U/mL), respectively. Other tests showed ALT levels at 55.00 U/L (reference range: 9–50 U/L) and AST levels at 81.50 U/L (reference range: 15–40 U/L). The patient was HBeAg-negative, and the HBV-DNA load was 5.325 × 10^3^ IU/mL (reference range: <10 IU/mL). Abdominal CT found an intrahepatic lesion measuring 18.1 × 13.1 cm (Figure 3A). In addition, liver MRI detected an intrahepatic lesion measuring 11.8 × 13.5 cm (Figure 3B). The clinical diagnosis was conducted in strict accordance with the latest EASL Clinical Practice Guidelines, based on hallmark features such as arterial phase hyperenhancement and rapid washout (LI-RADS 5). Ultimately, the patient was diagnosed with China Liver Cancer (CNLC) stage IIIa, according to the Guidelines for the Diagnosis and Treatment of Primary Liver Cancer (2024 edition). Unlike the Barcelona Clinic Liver Cancer (BCLC) staging system, the CNLC classification offers more detailed stratification and is widely adopted in China to guide multidisciplinary treatment.

Case 2 underwent transcatheter arterial chemoembolization (TACE) as the primary therapeutic intervention immediately following the diagnosis of HCC in February 2025. The procedure was successfully performed without any immediate complications. After discharge, the patient strictly adhered to the prescribed medications and reported no significant discomfort. One month later (March 2025), follow-up CT imaging revealed that the tumor had shrunk, with its maximal cross-section reduced to 10.8 × 9.6 cm; however, the tumor exhibited heterogeneous arterial enhancement (Figure 3C). Serum levels of AKR1B10 transiently increased to 3799.12 pg/mL due to the release from necrotic tumor cells. Serum AFP levels remained persistently negative at 3.25 ng/mL. The patient subsequently underwent a second TACE procedure.

In April 2025, the tumor further decreased in size to 9.3 × 8.4 cm, and serum AKR1B10 levels declined to 776.22 pg/mL, confirming the therapeutic response. The patient remained under close surveillance and underwent a third TACE procedure. During the follow-up in July 2025, imaging showed that the main tumor had further reduced to 8.3 × 8.1 cm, with increased iodized oil deposition (Figure 3D). Serum AFP levels remained persistently negative (2.38 ng/mL). The patient subsequently underwent a fourth TACE procedure.

**Figure 4:**
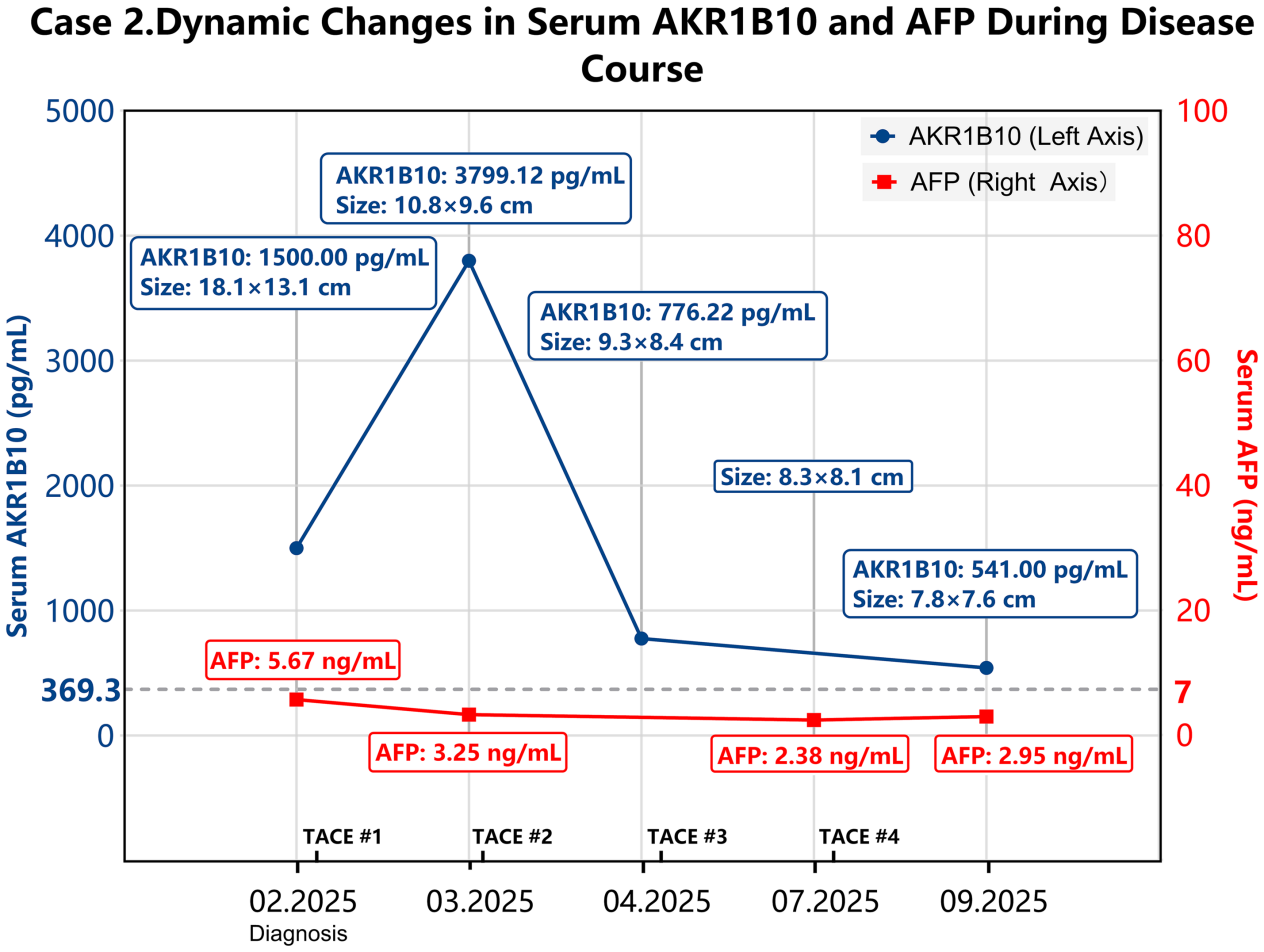
Chronological clinical events and dynamic changes in tumor size and serum AKR1B10 and AFP levels in Case 2. Serum AKR1B10 (blue line) is plotted on the left axis, while AFP (red line) is plotted on the right axis. Tumor size and TACE treatments are indicated. TACE, transcatheter arterial chemoembolization.

In September 2025, the tumor further decreased to 7.8 × 7.6 cm, and serum AKR1B10 levels declined to 541.00 pg/mL, indicating the effectiveness of TACE. Serum AFP levels remained consistently negative at 2.95 ng/mL. However, this admission was marked by typical signs of decompensated liver function, including hepatic facies, scleral and cutaneous jaundice, and palmar erythema; therefore, TACE was not conducted this time. Figure 4 shows the chronological events and dynamic changes in tumor imaging and serum AKR1B10 levels. Serum AFP levels remained negative throughout the procedures.

## 3 Discussion and conclusion

The diagnosis was missed in both cases at the early stage of the disease. Notably, Case 1 had annual health check-ups but was unfortunately misdiagnosed as having an atypical hepatic hemangioma. Early HCC is often asymptomatic, and serum AFP serves as an important indicator for early detection in patients with chronic hepatitis and cirrhosis. However, AFP remains persistently negative in a substantial portion of HCC patients, particularly in early-stage HCC, highlighting the diagnostic challenge of AFP-negative HCC. In these two cases, serum AFP levels were normal, while AKR1B10 levels were markedly elevated to 6239.29 pg/mL (Case 1) and 1500.00 pg/mL (Case 2). These findings underscore the potential utility of AKR1B10 in detecting AFP-negative HCC.

Early-stage HCC may be curable through radical resection, with a 5-year survival rate of 69.0–86.2%; however, patients with advanced HCC have a 5-year survival rate of <5.0% (12). Unfortunately, the majority of HCC patients are still diagnosed at intermediate and advanced stages. Therefore, it is critical to develop novel strategies for the screening and early detection of HCC to improve prognosis. Serum tumor markers are convenient, safe, and non-invasive, serving as important indicators for the dynamic monitoring and early detection of HCC. AFP is the most widely used tumor marker in HCC cases, but as indicated in these two cases, its limitations in HCC diagnosis are clearly evident. Data have shown that even using the most effective cutoff range of 0–20 ng/mL, the sensitivity of AFP is 60% (95%CI: 55–64%), with a specificity of 80% (13–15). Combining AFP with abdominal ultrasound may improve the detection of liver cancer, but the improvement is incremental (16).

Serum AKR1B10 is a recently identified marker for HCC. Clinical studies have shown its advantages in the detection of HCC, especially early-stage HCC, compared to AFP (17, 18). AKR1B10 also shows prognostic value in HCC patients at an early stage with low tumor burden and good liver function (18). The clinical course of Case 1 highlights a critical diagnostic pitfall. The misdiagnosis of the 2.3 cm lesion as an atypical hemangioma did not adhere to the EASL and AASLD guidelines, which would otherwise require rigorous dynamic imaging or biopsy for such nodules in HBV patients, regardless of serology. Nevertheless, this radiological error appeared to be reinforced by the persistently negative serum AFP levels, which may have inadvertently supported the benign diagnosis. As a result, this could have diminished the clinical justification for further challenging the imaging findings with an invasive biopsy. It is currently unknown whether AKR1B10 would increase during the early monitoring of HCC due to the lack of direct serological evidence; however, it is reasonable to postulate that serum AKR1B10 may have been helpful, given its sensitivity in detecting early-stage HCC (7).

In Case 2, serological monitoring of AKR1B10 was also missed before the diagnosis of HCC, but serial monitoring during interventional therapy revealed dynamic changes in serum AKR1B10 in response to TACE. At the time of diagnosis, serum AKR1B10 levels were elevated to 1500.00 pg/mL, whereas AFP levels were within the normal range. One month after the first interventional therapy, AKR1B10 levels increased to 3799.12 pg/mL, and imaging revealed a marked reduction in tumor size. This scenario may indicate the effectiveness of TACE, in which the cell death of HCC induced by the therapy leads to the release of AKR1B10 into the bloodstream. Supporting this interpretation, AKR1B10 levels decreased to 776.22 pg/mL, and the tumor further shrank to 9.3 × 8.4 cm 1 month after the second TACE therapy. Thereafter, serum AKR1B10 levels continued to decrease, and the tumor further shrank with additional TACE procedures (Figure 4). Unfortunately, the patient discontinued TACE due to liver function deterioration. Nevertheless, the chronological data from Case 2 revealed a consistent correlation between serum AKR1B10 levels, therapeutic efficacy, and tumor shrinkage. Notably, serum AFP levels remained persistently within the normal range throughout the entire course.

In these two cases, the elevated levels of serum AKR1B10, alongside persistently negative AFP levels, suggest that these two serum markers may reflect distinct biological processes in HCC. AFP is a differentiation-associated oncofetal glycoprotein whose expression largely depends on the reactivation of fetal-like transcriptional programs in hepatocytes. Previous studies have indicated that a substantial subset of HCC cases, including biologically aggressive tumors, may remain AFP-negative throughout their clinical course, indicating the limitation of AFP as a universal marker for HCC (19). To date, evidence indicates that AFP-negative HCC is characterized by alterations in cellular differentiation status, epigenetic regulation, and ultrastructural morphology, rather than being determined solely by tumor burden or disease stage (20–22). In these tumors, the fetal-like transcriptional programs required for AFP expression may not be fully reactivated, resulting in low or undetectable serum AFP levels even in advanced stages of the disease. In contrast, AKR1B10 is a cytosolic protein involved in lipid synthesis, detoxification of reactive carbonyl compounds, and cellular defense against oxidative stress (23). Therefore, AKR1B10 may support the proliferation and survival of tumor cells by mitigating lipid peroxidation-induced damage and facilitating metabolic adaptation under stressful microenvironmental conditions (24, 25). This biological distinction may explain the differing expression patterns of these two markers in HCC and supports the use of a combination of AKR1B10 and AFP for HCC detection.

In Case 2, serum AKR1B10 levels demonstrated dynamic fluctuations in response to TACE, highlighting its potential as a sensitive marker for HCC. In light of the remarkable shrinkage of the primary tumor mass, the transient increase in serum AKR1B10 levels after the first TACE procedure may be attributed to tumor cell death and the subsequent release of AKR1B10 into the bloodstream, thereby indicating the therapeutic efficacy. The subsequent decline in serum AKR1B10 levels after repeated interventions was correlated with further shrinking of the tumor mass, supporting its potential as a therapeutic marker for HCC. These findings underscore the need for a large-scale prospective clinical study.

It is noteworthy that, similar to all tumor markers, serum AKR1B10 levels may also increase in non-malignant liver conditions, such as liver cirrhosis, albeit to a much milder extent (7). Transcriptomic profiling in non-alcoholic steatohepatitis has indicated that oxidative stress may drive AKR1B10 expression in advanced fibrosis (26). However, unlike AFP, which is frequently false-positive during active hepatitis B flares, AKR1B10 maintains a comparatively stable profile (27), which may filter out inflammatory noise in AFP-positive patients. In addition, AKR1B10 offers advantages over AFP in differentiating HCC from benign liver tumors (17). Therefore, we recommend that serum AKR1B10 be used in combination with other serum markers, such as AFP and des-gamma carboxyprothrombin (DCP).

Considering the biological heterogeneity of HCC, discordant results between serum AFP and AKR1B10 may occasionally be observed in clinical practice. In such cases, clinical management should follow the fundamental principle that serum markers serve only as auxiliary diagnostic tools and that imaging tests, such as CT or MRI, remain essential for the diagnosis of HCC. In addition, the dynamic trends of serum markers are also important in the clinical diagnosis of HCC. A progressive or sustained increase in a serum marker should prompt timely escalation to enhanced imaging studies.

In summary, serum AFP has long been a widely used marker for HCC and remains an important test in clinical practice, despite its sensitivity and specificity. However, the diagnostic challenges observed in these two cases highlight the need for caution when interpreting the AFP results. The AKR1B10 data from these two cases provide hypothesis-generating evidence that AKR1B10 may serve as an adjunct marker in the diagnosis of AFP-negative HCC, warranting further investigation. This report also supports the rationale for using a combination of multiple serum markers, such as AKR1B10, AFP, and DCP, in HCC management.

## Data Availability

The original contributions presented in the study are included in the article/supplementary material, further inquiries can be directed to the corresponding authors.
